# Low serum albumin is an independent risk factor in elderly patients with aggressive B‐cell lymphoma: Results from prospective trials of the German High‐Grade Non‐Hodgkin's Lymphoma Study Group

**DOI:** 10.1002/jha2.61

**Published:** 2020-07-13

**Authors:** Karin Hohloch, Marita Ziepert, Lorenz Truemper, Christian Buske, Gerhard Held, Viola Poeschel, Bjoern Chapuy, Bettina Altmann

**Affiliations:** ^1^ Department of Hematology and Oncology Kantonsspital Graubünden Chur Switzerland; ^2^ Department of Hematology and Oncology University Medical Center Georg‐August University Göttingen Germany; ^3^ Statistics and Epidemiology Institute for Medical Informatics University of Leipzig Leipzig Germany; ^4^ Comprehensive Cancer Center Ulm Institute of Experimental Cancer Research University Hospital Ulm Ulm Germany; ^5^ Department of Internal Medicine University Hospital Saarland Homburg Germany

**Keywords:** aggressive lymphoma, elderly, low serum albumin, risk factor

## Abstract

Serum albumin a well‐known risk factor predicting outcome in many solid tumors. We explore the role of low serum albumin (≤3.5 g/dL) as an independent risk factor in elderly patients with aggressive B‐cell lymphoma. Outcome of 429 patients treated with R‐CHOP‐14 in the RICOVER‐60 trial and available serum albumin were analyzed in this retrospective study. Of the 429 patients in the RICOVER‐60 trial, 137 (32%) had low and 292 (68%) had normal serum albumin levels (>3.5 g/dL). In the low albumin group, patients had significantly higher International Prognostic Index (IPI), bulky disease, extralymphatic involvement, and B‐symptoms. Event‐free survival (EFS) (*P* < .001), progression‐free survival (PFS) (*P* < .001), and overall survival (OS) (*P* < .001) were significantly inferior for patients with low compared to those with normal serum albumin. Multivariate analysis adjusted for IPI shows following Hazard ratios (HR) for low serum albumin: EFS (HR = 1.5; 95% confidance interval [CI] [1.1; 2.1], *P* = .009), PFS (HR = 1.7; 95% CI [1.2; 2.4], *P* = .001) and OS (HR = 1.6; 95% CI [1.1; 2.3], *P* = .006). Results were confirmed in 185 patients from the DENSE‐R‐CHOP‐14 and SMARTE‐R‐CHOP‐14 trials. In conclusion, low serum albumin is an independent risk factor in elderly patients with aggressive B‐cell lymphoma treated with R‐CHOP.

## 1 INTRODUCTION

Albumin is an important serum protein in humans that maintains the oncotic pressure in the plasma and serves as carrier protein for hydrophobic molecules. Albumin is produced in the liver at a rate of 150‐250 mg/kg bodyweight accounting for 20% of the total protein synthesis capacity of the liver. Its hepatic synthesis is regulated by the oncotic pressure, inflammation, availability of amino acids, and to a lesser degree by nutritional status [1]. Albumin synthesis is stimulated by thyroxin, glucocorticoids, and anabolic steroids. Catabolism for albumin occurs like for other serum proteins in many tissues, particularly by pinocytosis in the vascular endothelium [2].

Hypoalbuminemia and malnourishment are often described in patients with cancer and reasons for it are not fully understood. In clinical practice, anthropometric methods or laboratory markers (hepatic proteins such as albumin, prealbumin, transferrin) are used for detection of malnourishment [3]. Among those albumin is most often used because of its easy availability. For a long time, hypoalbuminemia was interpreted almost exclusively as marker for malnourishment [1]. In cancer patients, release of proinflammatory cytokines (IL‐6, TNF) and catabolic factors may also play a more important role for these findings [4]. An increased vascular permeability with loss of albumin to the extravascular compartment might also contribute in cancer patients [5]. The degree of a perturbed albumin turnover in cancer or lymphoma patients is controversial. Two studies described impaired albumin synthesis with a normal survival in cancer including leukemia and lymphoma patients [6, 7], whereas another group described normal albumin synthesis rates in cachectic individuals with pancreatic cancer [8]. In cancer patients, malnourishment and low serum albumin prior to anticancer therapy is a well‐established prognostic marker. Low serum albumin was shown to be an independent prognostic factor for survival and prognosis in many solid tumors and Hodgkin lymphoma [9, 10, 11, 12, 13, 14, 15].

To gain insights into the prognostic role of pretreatment albumin serum levels, we analyzed 429 patients with aggressive B‐cell lymphoma treated with six or eight cycles of CHOP‐14 and eight applications of rituximab from the prospective, multicenter RICOVER‐60 trial [16] with respect to outcome following stratification of patients based on their serum albumin level.

## 2 MATERIALS AND METHODS

### 2.1 Patients and treatment

We identified 610 elderly R‐CHOP‐14 treated patients (61–80 years of age) with aggressive CD20^+^ B‐cell lymphoma included in the RICOVER‐60 trial [16]. The RICOVER‐60 [16] trial compared six or eight cycles of CHOP‐14 with or without eight applications of rituximab in 1222 elderly patients with aggressive B‐cell lymphoma (DLBCL, grade 3 follicular lymphoma, lymphoblastic lymphoma, blastoid mantle cell lymphoma, Burkitt lymphoma, and aggressive marginal zone lymphoma). Patients with bulky disease (≥7.5 cm) or extralymphatic involvement received consolidative involved field (IF) radiation with 36 Gy. From 610 R‐CHOP‐14 treated patients, 429 patients (70%) for whom total protein and serum albumin prior to therapy was available were included in the following analysis. As validation set, we analyzed data of 313 patients (124 patients out of the DENSE‐R‐CHOP‐14 [18] study, 189 patients out of the SMARTE‐R‐CHOP‐14 study [17, 18]) treated with 6xCHOP‐14 and 12 (DENSE‐R) or 8 (SMARTE‐R) applications rituximab. The DENSE‐R CHOP‐14 trial was an amendment of the RICOVER‐60 trial, the inclusion criteria for the SMARTE‐R trial were identical to the RICOVER‐60 trial (patients were eligible if they had previously untreated, biopsy‐confirmed aggressive non‐Hodgkin lymphoma of the B‐celltype according to WHO classification 11and were between ages 61 and 80 years). For 185 patients albumin levels were available and were analyzed.

### 2.2 Measurements

Albumin levels were requested per protocol in the baseline documentation of the RICOVER‐60, the DENSE‐R‐CHOP‐14 and the SMARTE‐R‐CHOP‐14 trials. Total serum protein (g/dL) and serum albumin levels (percentage of total serum protein) were documented prior to therapy. For the following analysis, serum albumin was calculated in g/dL.

### 2.3 Statistical analysis

Event‐free survival (EFS) as the primary endpoint of the RICOVER‐60, the DENSE‐R‐CHOP‐14, and the SMARTE‐R‐CHOP‐14 trials was defined as the time from randomization/registration to disease progression, start of salvage treatment, additional (unplanned) treatments, relapse, or death of any cause. Progression‐free survival (PFS) was defined as time from randomization/registration to disease progression, relapse, or death of any cause. Overall survival (OS) was defined as the time from randomization/registration to death of any cause. EFS, PFS, and OS were estimated according to Kaplan and Meier [19].

Before the analysis, Kaplan–Meier plots were created for EFS, PFS, and OS (RICOVER‐60) with respect to the four quartile groups of serum albumin showing a very poor prognosis for the quartile group with the lowest albumin levels (Supplement Figure 2). Furthermore, a martingale residual analysis was performed to check, whether the cutoff (≤ 3.5 g/dL) for albumin used in the literature is plausible for the analyzed data from the RICOVER‐60 trial and this was the case (figures not shown).

Univariable outcome analyses were performed using the cutoff for albumin ≤3.5 g/dL. Differences between survival curves were tested using log‐rank tests. Proportional hazard models were adjusted for the factors of the International Prognostic Index (IPI; ie, age > 60 years, lactate dehydrogenase (LDH) > normal, Eastern Cooperative Oncology Group performance score (ECOG PS) > 1, stage III/IV, and extralymphatic involvement > 1) and additionally, in a further analysis, for gender, age > 70 years, bulky disease, bone marrow involvement, B‐symptoms, and obesity (BMI < 30 vs ≥ 30 kg/m²). Hazard ratios (HR) with 95% confidence intervals (CI) and *P*‐values are presented. For comparison of patient characteristics, chi‐square and, if necessary, Fisher's exact tests were used. The significance level was *P* = .05 (two‐sided). Statistical analyses were performed with IBM SPSS Statistics 24 software (SPSS, Chicago, IL).

## 3 RESULTS

### 3.1 Clinical characteristics and outcome

In total, we analyzed 610 patients from the randomized, multicenter RICVOVER‐60 trial [16], treated with six or eight cycles of CHOP‐14 and 8 applications of rituximab. Four hundred twenty‐nine (70%) out of these 610 patients had available pretreatment serum albumin levels. To rule out a bias between the patients with available and nonavailable serum albumin levels, we compared baseline characteristics between the patient cohorts and could not find statistical differences in the distribution of the baseline characteristics (Supplement Table 1) or according EFS, PFS, and OS (Supplement Figure 1). Of these 429 patients, 233 (54%) were male and 196 (46%) were female. The median age was 69 with a range of 61‐80 years. 37% were above 70 years and 13% were older than 75 years. 31% of patients had an IPI of 1, 28% an IPI of 2, 25% an IPI of 3, and 15% an IPI of 4 or 5. Extralymphatic involvement was seen in 54% and bulky disease in 38% of the patients. Seventy‐nine percent of the patients were diagnosed with a diffuse large B‐cell lymphoma (DLBCL) and 19% with other B‐cell lymphomas (Supplement Table 1). One hundred thirty‐seven (32%) of the patients had a low serum albumin (≤3.5 g/dL), and 292 (68%) had a serum albumin within normal limits (>3.5 g/dL). In the low albumin group, there were significantly more patients with higher IPI risk factors (LDH > normal, ECOG > 1, stage III/IV, extralymphatic involvement > 1), extralymphatic involvement, bulky disease, B‐symptoms, and lower body mass index (BMI) (Table 1). The clinical characteristic according albumin levels for the 185 patients from the DENSE‐R‐CHOP‐14 [18] and the SMARTE‐R‐CHOP‐14 [16] trial validation cohort is comparable with the RICOVER‐60 trial (Table 1, Supplement Table 1, Supplement Figure 1).

**TABLE 1:** Clinical characteristics of patients with albumin value ≤ 3.5 g/dL and patients with albumin value > 3.5 g/dL

	RICOVER‐60			DENSE‐R/SMARTE‐R‐CHOP‐14		
	Albumin ≤3.5 g/dL(*n* = 137)	Albumin>3.5 g/dL (*n* = 292)	*P*‐value	Albumin ≤3.5 g/dL(*n* = 52)	Albumin>3.5 g/dL (*n* = 133)	*P*‐value
Male	68 (50%)	165 (57%)	.183	33 (63%)	63 (47%)	.049
Female	69 (50%)	127 (43%)		19 (37%)	70 (53%)	
Age, median (range)	69 (61,80)	68 (61,80)	.093	70 (61,80)	68 (61,80)	.091
Age > 60 and ≤ 65	39 (28%)	94 (32%)	.343	9 (17%)	38 (29%)	.390
Age > 65 and ≤ 70	41 (30%)	95 (33%)		18 (35%)	45 (34%)	
Age > 70 and ≤ 75	33 (24%)	70 (24%)		14 (27%)	30 (23%)	
Age > 75 and ≤ 80	24 (18%)	33 (11%)		11 (21%)	20 (15%)	
LDH > N	85 (62%)	122 (42%)	<.001	41 (79%)	63 (47%)	<.001
ECOG > 1	38 (28%)	21 (7%)	<.001	13 (25%)	13 (10%)	.007
Stage III / IV	88 (64%)	118 (40%)	<.001	43 (83%)	83 (62%)	.008
E > 1	39 (28%)	33 (11%)	<.001	23 (44%)	37 (28%)	.032
IPI 1	22 (16%)	113 (39%)	<.001	5 (10%)	36 (27%)	.001
IPI 2	32 (23%)	90 (31%)		8 (15%)	30 (23%)	
IPI 3	44 (32%)	65 (22%)		14 (27%)	40 (30%)	
IPI 4, 5	39 (28%)	24 (8%)		25 (48%)	27 (20%)	
E‐involvement	91 (66%)	139 (48%)	<.001	40 (77%)	81 (61%)	.039
Bulky disease	73 (53%)	88 (30%)	<.001	21 (40%)	36 (27%)	.078
B‐symptoms	66 (48%)	68 (23%)	<.001	22 (42%)	29 (22%)	.005
Bone marrow involved	12 (9%)	10 (3%)	.020	7 (13%)	14 (11%)	.572
BMI^a^(kg/m²) < 18.5	1 (1%)	4 (1%)	<.001			
BMI (kg/m²) ≥ 18.5 & < 25	66 (52%)	80 (29%)				
BMI (kg/m²) ≥ 25 & < 30	39 (31%)	138 (50%)				
BMI (kg/m²) ≥ 30	21 (17%)	53 (19%)				
Reference pathology^a^ :						
DLBCL	102 (77%)	228 (79%)		42 (82%)	105 (80%)	
Follicular lymphoma III°	6 (5%)	18 (6%)		0 (0%)	6 (5%)	
Follicular lymphoma III° + DLBL	5 (4%)	10 (3%)		6 (12%)	8 (6%)	
Lymphoblastic precursor B‐cell lymphoma	0 (0%)	1 (0.3%)		0 (0%)	0 (0%)	
Burkitt‘s lymphoma	2 (2%)	4 (1%)		0 (0%)	0 (0%)	
Burkitt‐like	0 (0%)	1 (0.3%)		0 (0%)	0 (0%)	
Mantle cell lymphoma (blastic)	3 (2%)	5 (2%)		0 (0%)	1 (1%)	
Mantle cell lymphoma, classical type	0 (0%)	1 (0.3%)		0 (0%)	0 (0%)	
Aggressive marginal zone lymphoma	4 (3%)	2 (1%)		0 (0%)	0 (0%)	
B‐cell, NOS	4 (3%)	4 (1%)		1 (2%)	1 (1%)	
B‐cell, unclassified (technical insufficient material)	4 (3%)	5 (2%)		2 (4%)	5 (4%)	
Low grade NHL	2 (2%)	6 (2%)		0 (0%)	5 (4%)	
Hodgkin‘s disease	0 (0%)	1 (0.3%)		0 (0%)	0 (0%)	
No lymphoma	0 (0%)	1 (0.3%)		0 (0%)	0 (0%)	

a some missing values.

### 3.2 Low serum albumin and outcome

**FIGURE 1:**
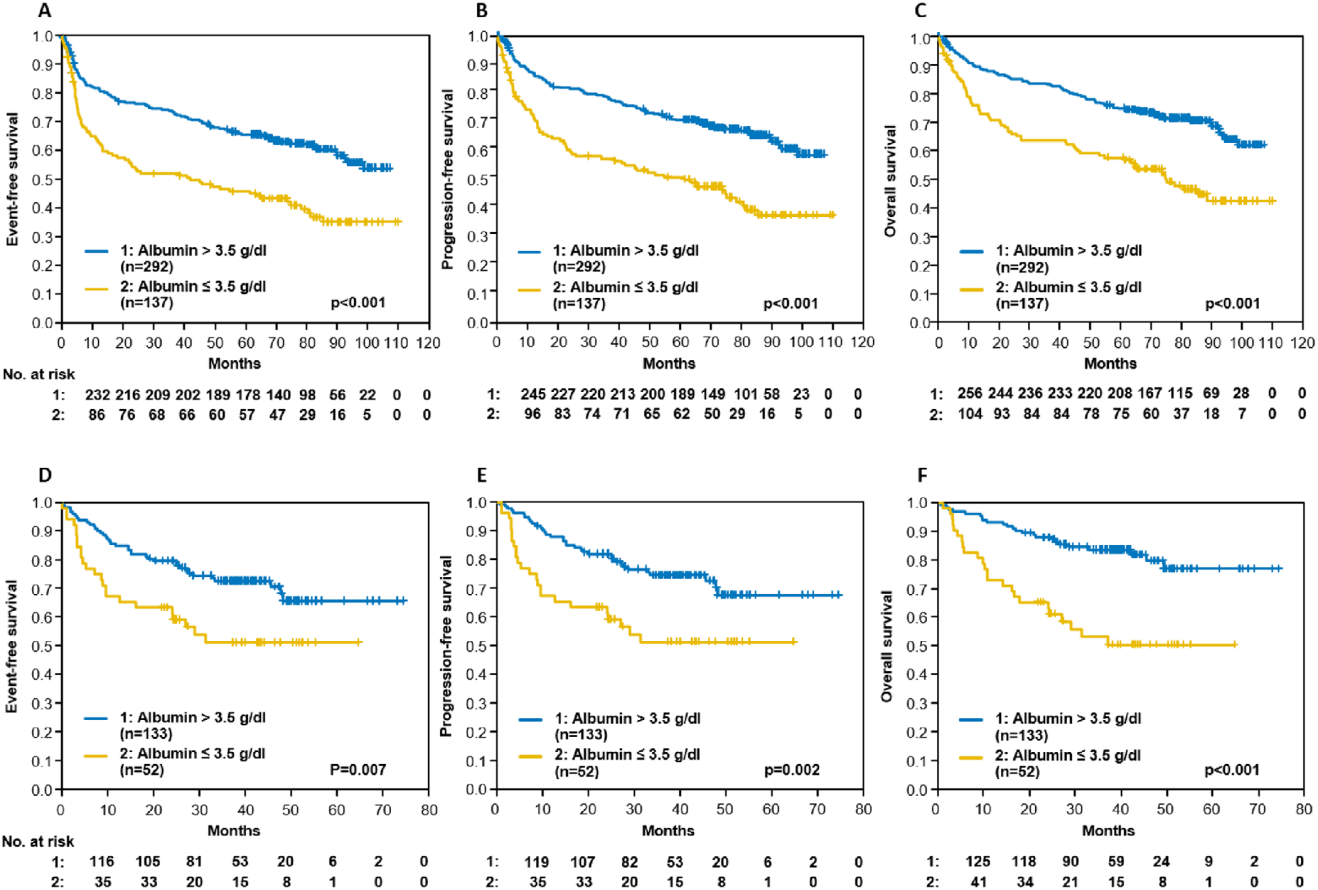
Event‐free, progression‐free and overall survival according to serum albumin (≤ 3.5 g/dL vs > 3.5 mg/dL). A‐C: RICOVER‐60 trial; D‐F: DENSE‐R‐CHOP‐14/SMARTE‐R‐CHOP‐14 trials

Notably, EFS (*P* < .001), PFS (*P* < .001), and OS (*P* < .001) were significantly inferior for patients with low serum albumin (≤3.5 g/dL) compared to those with normal serum albumin (Figure 1A‐C). In a multivariate analysis adjusting for IPI risk factors (LDH > N; stage III/IV, ECOG > 1, > 1 extralymphatic involvement), these results were confirmed to be predictive for the respective clinical endpoints with the following hazard ratios (HR) for low serum albumin: EFS (HR = 1.5; 95%CI [1.1; 2.1], *P* = .009), PFS (HR = 1.7; 95% CI [1.2; 2.4], *P* = .001), and OS (HR = 1.6; 95%CI [1.1; 2.3], *P* = .006) (Table 2). These results were also consistent after additional adjustment for well‐known risk factors or different distributions, seen in the clinical characteristic, for example, male gender, age > 70 years, BMI ≥ 30, B‐symptoms, bone marrow involvement, and bulky disease (data not shown). Furthermore, EFS, PFS, and OS is significant separated by albumin levels within the IPI group 1, 2, and within the IPI group 3‐5 (Supplement Figure 3). These results were validated and confirmed within 185 patients from the DENSE‐R‐CHOP‐14 and the SMARTE‐R‐CHOP‐14 trials. In this validation set, patients with low serum albumin had a significantly inferior EFS (*P* = .007), PFS (*P* = .002), and OS (*P* < .001) (Figure 1D‐F). HR for low serum albumin in the multivariate analysis adjusting for IPI risk factors were significantly elevated: EFS (HR = 1.9; 95%CI [1.1; 3.2], *P* = .023), PFS (HR = 2.1; 95% CI [1.2; 3.7], *P* = .009), and OS (HR = 3.1; 95%CI [1.7; 5.7], *P* < .001) (Table 2). The results were also confirmed when evaluating exclusively the patients with DLBCL in the RICOVER‐60 trial (Supplement Table 2, supplement Figure 4).

**TABLE 2:** Multivariate analysis of event‐free (EFS), progression‐free (PFS) and overall survival (OS) adjusting for IPI factors for patients from the RICOVER‐60 trial and for patients from the DENSE‐R/SMARTE‐R‐CHOP‐14 trials, respectively

	EFSHR (95% CI)	*P*	PFSHR (95% CI)	*P*	OSHR (95% CI)	*P*
RICOVER‐60 (*n* = 429)						
Albumin ≤ 3.5 vs > 3.5 g/dL	1.5 (1.1‐2.1)	.009	1.7 (1.2‐2.4)	.001	1.6 (1.1‐2.3)	.006
LDH > N	1.5 (1.1‐2.1)	.006	1.6 (1.2‐2.3)	.003	1.7 (1.2‐2.4)	.002
ECOG > 1	1.3 (0.9‐1.9)	.227	1.2 (0.8‐1.8)	.471	1.3 (0.8‐1.9)	.264
Stage III/IV	1.4 (1.0‐1.9)	.066	1.3 (0.9‐1.9)	.163	1.3 (0.9‐1.9)	.175
E > 1	1.1 (0.8‐1.7)	.492	1.2 (0.8‐1.9)	.292	1.3 (0.9‐2.0)	.180
DENSE‐R/SMARTE‐R (*n* = 185)						
Albumin ≤ 3.5 vs > 3.5 g/dL	1.9 (1.1‐3.2)	.023	2.1 (1.2‐3.7)	.009	3.1 (1.7‐5.7)	< .001
LDH > N	0.9 (0.5‐1.5)	.594	0.8 (0.4‐1.4)	.444	0.8 (0.4‐1.7)	.629
ECOG > 1	1.2 (0.6‐2.4)	.631	1.3 (0.6‐2.6)	.488	1.6 (0.8‐3.4)	.191
Stage III/IV	1.8 (0.9‐3.6)	.088	1.9 (0.9‐3.9)	.073	1.4 (0.7‐3.0)	.384
E > 1	0.9 (0.5‐1.6)	.690	0.9 (0.5‐1.6)	.627	0.8 (0.4‐1.5)	.427

### 3.3 Low serum albumin and molecular subgroups

In a recent publication, a comprehensive genomic analysis identified five genetically defined DLBCL subgroups (C1‐C5 DLBCLs). The genetic signature of each DLBCL subtype provided insights into prognosis, suggested a different lymphomagenesis and suggested targeted treatments [20, 21]. Due to the small sample size for which the genomic analysis and albumin data was available, this additional analysis is restricted to 93 RICOVER‐60 data sets. For analysis, the patients were grouped in the recently defined genetic DLBCL subtypes C0/C1/C4: < = 3.5 (13 pts.) vs > 3.5 (21 pts.); C2 < = 3.5 (3 pts.) vs > 3.5 (14 pts.), and C3/C5 < = 3.5 (14 pts.) vs > 3.5 (28 pts.) as suggested in the original paper [20]. Nevertheless, it is notable that the multivariate analysis found low albumin to be still a significant independent prognostic factor adjusting for genetically defined DLBCL subtypes (C0/C1/C4; C2; C3/C5) and for the IPI risk factors (PFS: HR = 3.0, 95% CI [1.4; 6.4], *P* = .003).

## 4 DISCUSSION

Predicting outcome the IPI is still the most powerful prognostic score for aggressive B‐cell lymphoma. In the initial report of the IPI, pretreatment serum albumin levels were not included in the stepdown regression analysis due to insufficient data [22]. Our study demonstrated that low serum albumin prior to therapy is an independent unfavorable risk factor in elderly patients with aggressive B‐cell lymphoma. These results are in accordance with several smaller, retrospective studies showing that low serum albumin is a prognostic factor for survival in patients with DLBCL treated with R‐CHOP [23, 24, 25, 26]. The French prospective study of Peyrade et al. [27], a multicentre, phase II, single arm trial with an attenuated R‐mini CHOP regimen in 150 elderly patients (> 80 years) with DLBCL showed for the first time that low serum albumin is associated with a shorter overall survival. In this trial, low serum albumin ≤3.5 g/dL was the only factor affecting overall survival in the multivariate analysis (HR 3.2, 95% CI [1.4‐7.1]; *P* = .0053) [27]. In addition to the published data, our analysis presents a well‐defined study population including male and female patients treated in prospective randomized trials in the rituximab era. Treatment was standardized within a well‐defined dose‐dense protocol (R‐CHOP‐14) including standard supportive treatment and dose reduction strategies for all patients.

In our study, serum albumin levels were documented per protocol prior to therapy and were available for 70% of patients. Elderly [28], male [29], or malnourished patients [15] with DLBCL are known to have a inferior prognosis. In univariate and in multivariate analysis adjusted for IPI factors, gender, age > 70 years and all the factors with a significant difference between the low and normal serum albumin group (bulky disease, B‐Symptoms, or BMI), albumin remained an independent prognostic factor for survival. Therefore, a bias regarding these factors, especially age and gender, can be ruled out. However, patients with low serum albumin levels prior to start of therapy showed a significantly higher proportion of factors associated with worse prognosis and outcome indicating a more aggressive disease. A key question is, if low serum albumin levels in these patients are a consequence or one of the reasons for the more aggressive course of the lymphoma with impaired outcome.

To date the reason for hypoalbuminemia in patients with cancer or lymphoma is still not fully understood. Malnourishment does not seem to play a pivotal role as Jeevanandam et al. showed that protein turnover and protein metabolism is much higher in cancer patients than in individuals without cancer with the same defined caloric intake [30]. These data supported the concept that higher turnover is the cause of the hypoalbuminemia and not malnourishment. Although newer publications indicate that severe hypoalbuminemia in real malnourishment like kwashiorkor takes several weeks to month to arise and hypoalbuminemia in the critically ill patients is therefore not caused by malnourishment rather provoked by the inflammatory state in these patients [1]. In patients with aggressive lymphoma, onset of the disease is quite fast and patients are critically ill within days to a few weeks therefore “real” malnourishment is unlikely. The literature is controversial about reduced albumin synthesis or degradation in cancer and lymphoma patients. Albumin is a negative acute phase protein; therefore, release of proinflammatory cytokines (eg, IL‐6, TNF) leads to reduced albumin synthesis in the liver and to hypoalbuminemia in cancer patients [6, 7]. This finding is confirmed by an in vitro study in hepatocytes [31]. To the contrary, a small study in hypoalbuminemic, cachectic patients with pancreatic cancer found a normal albumin synthesis rate [8]. Other explanations for hypoalbuminemia in cancer patients include a higher vascular permeability due to cytokines and a subsequent leaking of albumin in the extracellular compartment [5].

Recent work also highlights a role of a 16‐mer fragment of human albumin as an endogenous antagonist for the chemokine receptor CXCR4, also known as endogenous peptide inhibitor of CXCR4 [EPI‐X4] [32, 33]. Specifically, EPI‐X4 can be generated from an abundant albumin precursor by Cathepsin D and E [33]. Since CXCR4 plays a central role in B‐cell homing and cancer cell migration, it is intriguing to speculate that normal albumin levels might be necessary to generate sufficient EPI‐X4 levels to impair lymphoma migration. Indeed, we could recently show that EPI‐X4 is able to block intrinsic cell growth and migration of B‐NHL cells in vitro (Personal communication Prof. Christian Buske, January 2020), assigning albumin a key role for endogenous control of CXCR4 mediated B‐NHL growth.

Current efforts in improving prognostic models of DLBCL focus on capturing tumor intrinsic molecular and genetic heterogeneity of the disease [20, 34, 35, 36]. However, it suggests that the nontumor intrinsic factor albumin is a prognostic factor assessing the urgency of the elderly patient for immunochemotherapy, independent of its genetic makeup. This analysis was made only in elderly patients; however, an analysis in younger patients recruited into DSHNHL and GLA (German Lymphoma Alliance) trials is planned

## 5 CONCLUSION

Our study establishes in a large, well‐defined clinical trial population pretreatment serum albumin levels as a cheap and easily available independent prognostic factor for survival in elderly patients with aggressive B‐cell lymphoma in the R‐CHOP era. Further studies will be needed to develop albumin in the context of other emerging markers and understand its biological relevance. Since it is fast, easy available and provides access to a nontumor intrinsic metabolic factor it might help to identify high‐risk patients early‐on and may aid in providing improved diagnostic and therapeutic approaches in these patients.

## Supporting information

FigureS1

TableS1
