# Supplementary material for: Low serum albumin is an independent risk factor in elderly patients with aggressive B‐cell lymphoma: Results from prospective trials of the German High‐Grade Non‐Hodgkin's Lymphoma Study Group
Source: EJHaem. 2020 Jul 13;1(1):181–7. doi: 10.1002/jha2.61 (PMC9175786; doi:10.1002/jha2.61)

Supplement figure 1: Event-free, progression-free and overall survival according to availability of serum albumin value. A-C: RICOVER-60 trial; D-F: DENSE-R-CHOP-14/SMARTE-R-CHOP-14 trials.


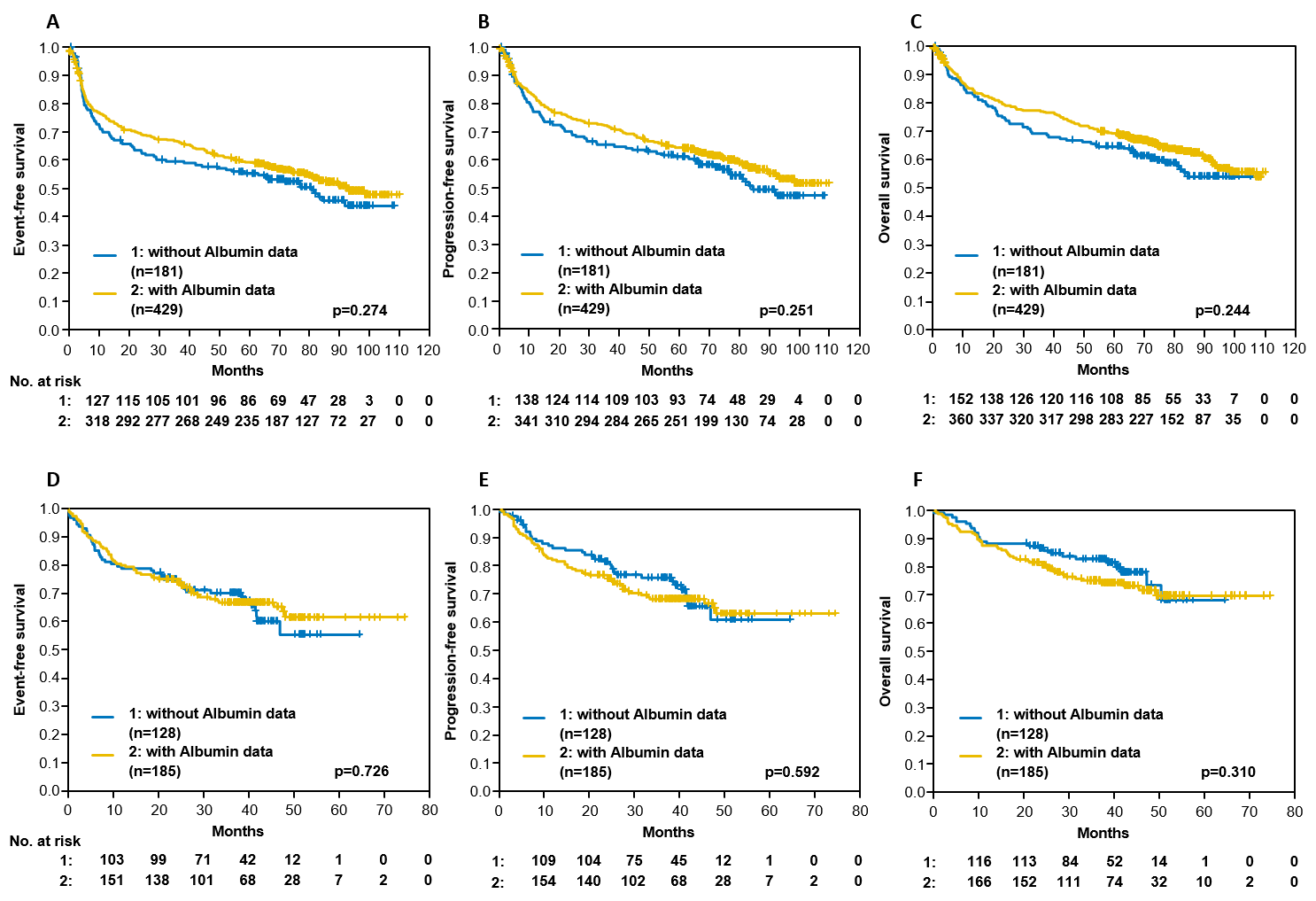


Supplement figure 2: Event-free (A), progression-free (B) and overall survival (C) of patients from the RICOVER-60 trial according to serum albumin quartile groups.


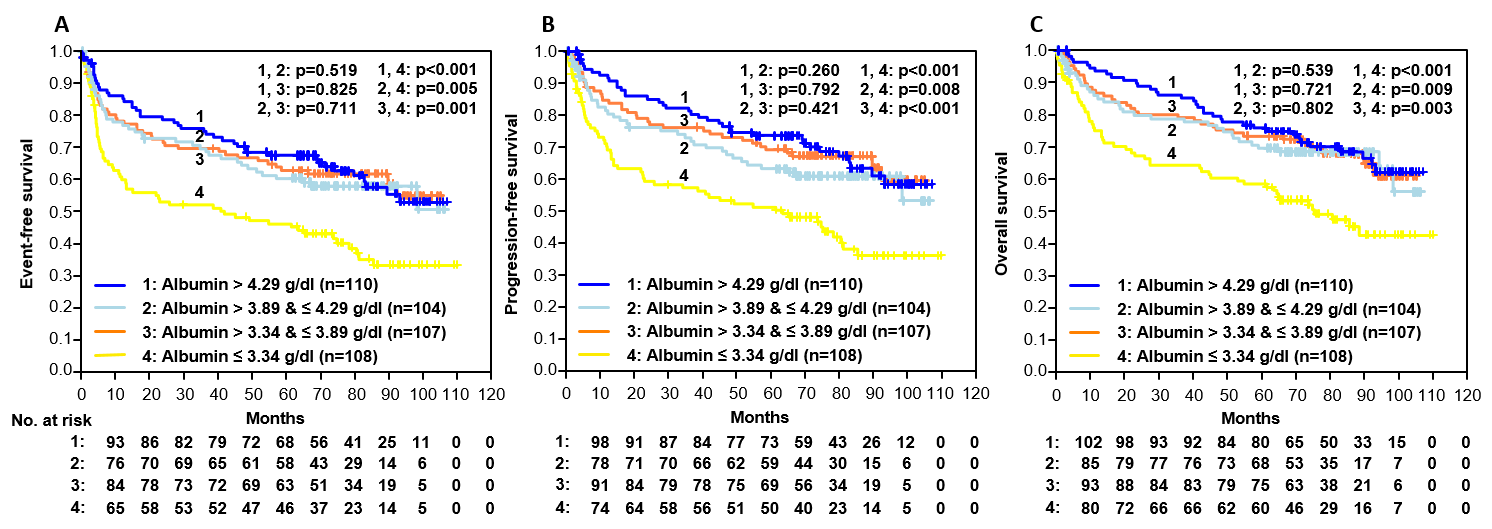


Supplement figure 3: Event-free (A), progression-free (B) and overall survival (C) of patients from the RICOVER-60 trial according to serum albumin (≤ 3.5 g/dl vs. > 3.5 mg/dl) within the IPI groups 1, 2 and 3-5.


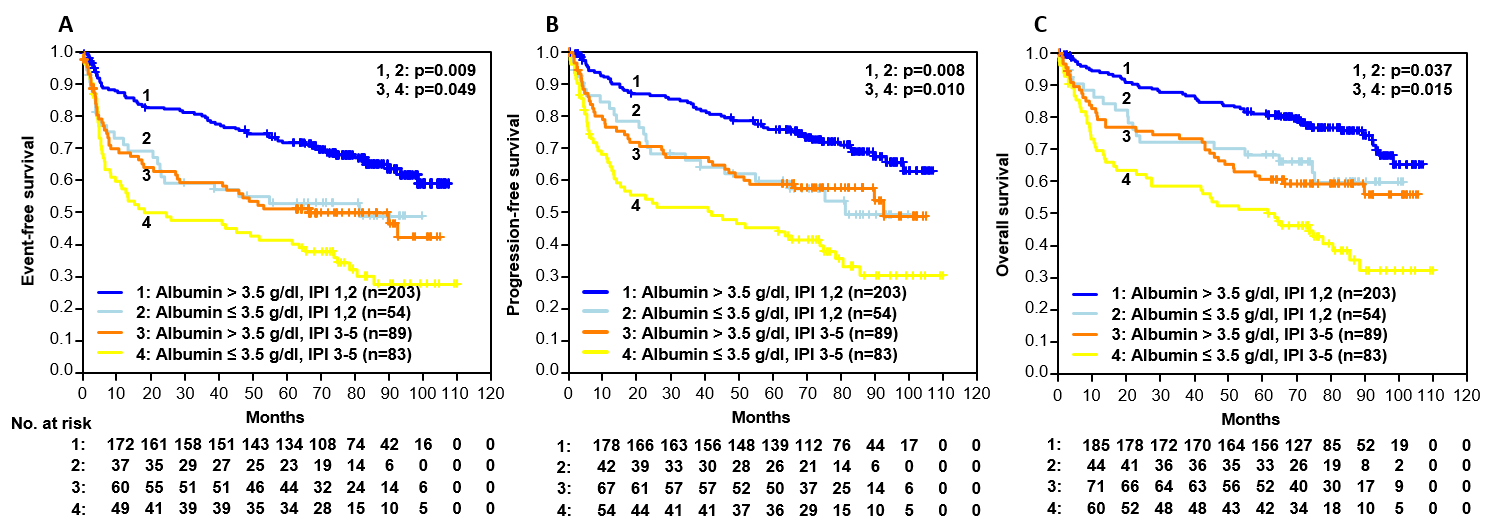


Supplement figure 4: Event-free (A), progression-free (B) and overall survival (C) of DLBCL patients from the RICOVER-60 trial according to serum albumin (≤ 3.5 g/dl vs. > 3.5 mg/dl).


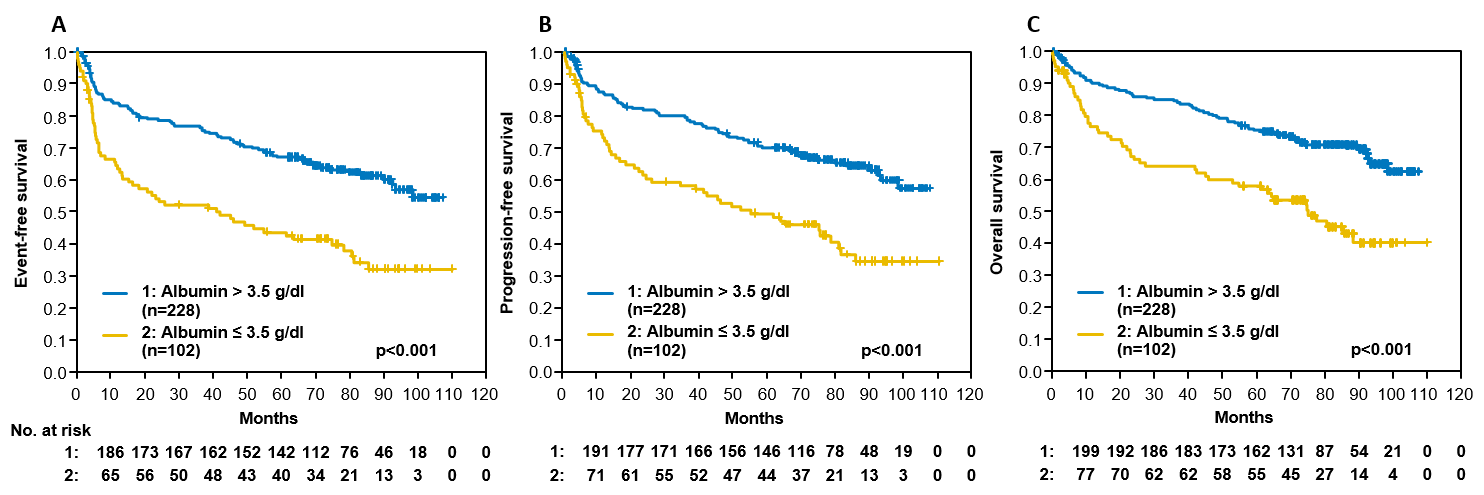

Supplement: Supplementary file 1 — FigureS1 [file JHA2-1-181-s002.docx]
