# Supplementary material for: Low serum albumin is an independent risk factor in elderly patients with aggressive B‐cell lymphoma: Results from prospective trials of the German High‐Grade Non‐Hodgkin's Lymphoma Study Group
Source: EJHaem. 2020 Jul 13;1(1):181–7. doi: 10.1002/jha2.61 (PMC9175786; doi:10.1002/jha2.61)
Supplement: Supplementary file 2 — TableS1 [file JHA2-1-181-s001.docx]

**Supplement table 1:**

**Clinical characteristics of patients without albumin data and patients with albumin data.**

|  | **RICOVER-60** | | | | | **DENSE-R/SMARTE-R-CHOP-14** | | | | |
| --- | --- | --- | --- | --- | --- | --- | --- | --- | --- | --- |
|  | **without Albumin data**  **(n=181)** | | **with Albumin data**  **(n=429)** | | **p-value** | **without Albumin data**  **(n=128)** | | **with Albumin data**  **(n=185)** | | **p-value** |
| Male  Female | 92  89 | (51%)  (49%) | 233  196 | (54%)  (46%) | 0.431 | 66  62 | (52%)  (48%) | 96  89 | (52%)  (48%) | 0.954 |
| Age, median (range) | 68 | (61,80) | 69 | (61,80) | 0.366 | 68 | (61,80) | 69 | (61,80) | 0.220 |
| Age > 60 & ≤ 65 | 65 | (36%) | 133 | (31%) | 0.696 | 37 | (29%) | 47 | (25%) | 0.864 |
| Age > 65 & ≤ 70 | 52 | (29%) | 136 | (32%) |  | 44 | (34%) | 63 | (34%) |  |
| Age > 70 & ≤ 75 | 41 | (23%) | 103 | (24%) |  | 29 | (23%) | 44 | (24%) |  |
| Age > 75& ≤ 80 | 23 | (13%) | 57 | (13%) |  | 18 | (14%) | 31 | (17%) |  |
| LDH > N | 96 | (53%) | 207 | (48%) | 0.280 | 65 | (51%) | 104 | (56%) | 0.343 |
| ECOG > 1 | 30 | (17%) | 59 | (14%) | 0.367 | 18 | (14%) | 26 | (14%) | 0.998 |
| Stage III / IV | 101 | (56%) | 206 | (48%) | 0.079 | 73 | (57%) | 126 | (68%) | 0.045 |
| E > 1 | 37 | (20%) | 72 | (17%) | 0.281 | 40 | (31%) | 60 | (32%) | 0.825 |
| IPI 1 | 49 | (27%) | 135 | (31%) | 0.398 | 31 | (24%) | 41 | (22%) | 0.465 |
| IPI 2 | 50 | (28%) | 122 | (28%) |  | 31 | (24%) | 38 | (21%) |  |
| IPI 3 | 46 | (25%) | 109 | (25%) |  | 40 | (31%) | 54 | (29%) |  |
| IPI 4, 5 | 36 | (20%) | 63 | (15%) |  | 26 | (20%) | 52 | (28%) |  |
| E-involvement | 98 | (54%) | 230 | (54%) | 0.904 | 87 | (68%) | 121 | (65%) | 0.637 |
| Bulky disease | 72 | (40%) | 161 | (38%) | 0.601 | 53 | (41%) | 57 | (31%) | 0.054 |
| B-symptoms | 63 | (35%) | 134 | (31%) | 0.389 | 39 | (30%) | 51 | (28%) | 0.577 |
| Bone marrow involved | 16 | (9%) | 22 | (5%) | 0.083 | 14 | (11%) | 21 | (11%) | 0.909 |
| BMI*(kg/m²) < 18.5 | 1 | (1%) | 5 | (1%) | 0.344 |  |  |  |  |  |
| BMI (kg/m²) ≥ 18.5 & < 25 | 74 | (43%) | 146 | (36%) |  |  |  |  |  |  |
| BMI (kg/m²) ≥ 25 & < 30 | 64 | (37%) | 177 | (44%) |  |  |  |  |  |  |
| BMI (kg/m²) ≥ 30 | 35 | (20%) | 74 | (18%) |  |  |  |  |  |  |
| **Reference pathology* :** |  |  |  |  |  |  |  |  |  |  |
| DLBCL | 144 | (83%) | 330 | (79%) | 0.567 | 107 | (84%) | 147 | (81%) | 0.660 |
| other B-cell | 27 | (16%) | 79 | (19%) |  | 16 | (13%) | 30 | (16%) |  |
| other | 3 | (2%) | 10 | (2%) |  | 4 | (3%) | 5 | (3%) |  |
|  |  |  |  |  |  |  |  |  |  |  |

* some missing values

**Supplement table 2:**

**Multivariate analysis of event-free (EFS), progression-free (PFS) and overall survival (OS) adjusting for IPI factors for DLBCL patients from the RICOVER-60 trial.**

|  | **EFS**  **HR (95% CI) p** | **PFS**  **HR (95% CI) p** | **OS**  **HR (95% CI) p** |
| --- | --- | --- | --- |
|  |  |  |  |
| **RICOVER-60**  **(n=330)** |  |  |  |
| Albumin ≤ 3.5 vs. > 3.5 g/dl | 1.8 (1.2-2.5) 0.002 | 1.7 (1.2-2.5) 0.004 | 1.6 (1.1-2.4) 0.011 |
| LDH > N | 1.6 (1.1-2.2) 0.010 | 1.7 (1.2-2.4) 0.006 | 1.8 (1.2-2.6) 0.005 |
| ECOG > 1 | 1.3 (0.8-2.0) 0.273 | 1.3 (0.8-2.0) 0.346 | 1.4 (0.9-2.3) 0.172 |
| Stage III / IV | 1.2 (0.8-1.8) 0.287 | 1.3 (0.8-2.0) 0.241 | 1.3 (0.8-2.0) 0.243 |
| E > 1 | 1.2 (0.8-1.9) 0.454 | 1.3 (0.8-2.1) 0.286 | 1.4 (0.9-2.3) 0.141 |
|  |  |  |  |
